# Management of Spinal Deformities and Evidence of Treatment Effectiveness

**DOI:** 10.2174/1874325001711011521

**Published:** 2017-12-29

**Authors:** Josette Bettany-Saltikov, Deborah Turnbull, Shu Yan Ng, Richard Webb

**Affiliations:** 1 Institute of Health and Social Care, Teesside University, Middlesbrough, UK; 2 Kingston-upon-Thames, Scoliosis UK Ltd. UK; 3 Wanchai Chiropractic Clinic, Hong Kong; 4 Peacocks Medical Group, Newcastle-upon-Tyne, Newcastle, UK

**Keywords:** Scoliosis, Adolescent idiopathic scoliosis, Adult scoliosis, Hyperkyphosis, Scheuermann’s disease, Rehabilitation, Exercises, Bracing, Surgery, PSSE, PSSR

## Abstract

**Introduction:**

The review evaluates the up-to-date evidence for the treatment of spinal deformities, including scoliosis and hyperkyphosis in adolescents and adults.

**Material and Methods:**

The PubMed database was searched for review articles, prospective controlled trials and randomized controlled trials related to the treatment of spinal deformities. Articles on syndromic scoliosis were excluded and so were the articles on hyperkyphosis of the spine with causes other than Scheuermann’s disease and osteoporosis. Articles on conservative and surgical treatments of idiopathic scoliosis, adult scoliosis and hyperkyphosis were also included. For retrospective papers, only studies with a follow up period exceeding 10 years were included.

**Results:**

The review showed that early-onset idiopathic scoliosis has a worse outcome than late-onset idiopathic scoliosis, which is rather benign. Patients with AIS function well as adults; they have no more health problems when compared to patients without scoliosis, other than a slight increase in back pain and aesthetic concern. Conservative treatment of adolescent idiopathic scoliosis (AIS) using physiotherapeutic scoliosis-specific exercises (PSSE), specifically PSSR and rigid bracing was supported by level I evidence. Yet to date, there is no high quality evidence (RCT`s) demonstrating that surgical treatment is superior to conservative treatment for the management of AIS. For adult scoliosis, there are only a few studies on the effectiveness of PSSEs and a conclusion cannot as yet be drawn.

For hyperkyphosis, there is no high-quality evidence for physiotherapy, bracing or surgery for the treatment of adolescents and adults. However, bracing has been found to reduce thoracic hyperkyphosis, ranging from 55 to 80° in adolescents. In patients over the age of 60, bracing improves the balance score, and reduces spinal deformity and pain. Surgery is indicated in adolescents and adults in the presence of progression of kyphosis, refractory pain and loss of balance.

**Discussion:**

The available evidence reviewed has suggested that different approaches are needed towards the management of different spinal deformities. Specific exercises should be prescribed in children and adolescents with a Cobb angle in excess of 15°. In progressive curves, they should be used in conjunction with bracing. Clarity regarding differences and similarities is given as to what makes PSSE and PSSR specific exercises. As AIS is relatively benign in nature, conservative treatment should be tried when the curve is at a surgical threshold, before surgery is considered. Similarly, bracing and exercises should be prescribed for patients with hyperkyphosis, particularly when the lumbar spine is afflicted. Surgery should be considered only when the symptoms cannot be managed conservatively.

**Conclusion:**

There is at present high quality evidence in support of the conservative treatment of AIS. The current evidence supports the use of PSSE, especially those using PSSR, together with bracing in the treatment of AIS. In view of the lack of medical consequences in adults with AIS, conservative treatment should be considered for curves exceeding the formerly assumed range of conservative indications.

There is, however a lack of evidence in support of any treatment of choice for hyperkyphosis in adolescents and spinal deformities in adults. Yet, conservative treatment should be considered first. Yet to date, there is no high quality evidence (RCT`s) demonstrating that surgical treatment is superior to conservative treatment for the management of AIS and hyperkyphosis. Additionally, surgery needs to be considered with caution, as it is associated with a number of long-term complications.

## 1 INTRODUCTION

The review evaluates the latest up-to-date evidence for the treatment of spinal deformities, including scoliosis and hyperkyphosis in adolescents and adults.

## 2 MATERIALS AND METHODS

The PubMed database was searched for review articles, Prospective Controlled Trials (PCT) with an untreated control group as well as Randomized Controlled Trials (RCT). Retrospective papers were only included when long-term results were published with a follow-up time exceeding 10 years. The reference lists cited in the reviews were also checked for any primary papers meeting the review criteria. Titles and abstracts were first screened after which a full paper review was performed. A hand search was also conducted where appropriate. The PICO acronym was used to search for specific terms related to all aspects of the research question. Population involved adolescents and adults with scoliosis or kyphosis. Intervention: all types of treatment, surgical and non-surgical. Comparative group: as above. Outcomes: cobb angle, posture, back pain, spinal mobility.

## 3 HISTORICAL CONTEXT OF SPINAL DEFORMITIES

Spinal deformities have been known for thousands of years. Reference to them is found as far back as prehistoric times, in the ancient Vedic mythological literature, where the spine was the symbolic equivalent of Mount Meru, the traditional center of the universe [1]. Mention of spinal deformities is also found in the Edward Smith Papyrus, which relates the illnesses and injuries of those who built the great pyramids in the 25th century B.C [1]. The Bible also contains passages where reference is made to crooked backed individuals who were “forbidden from offering sacrifices to the Lord” [Bible, 21st chapter of Leviticus].

**Fig. (1):**
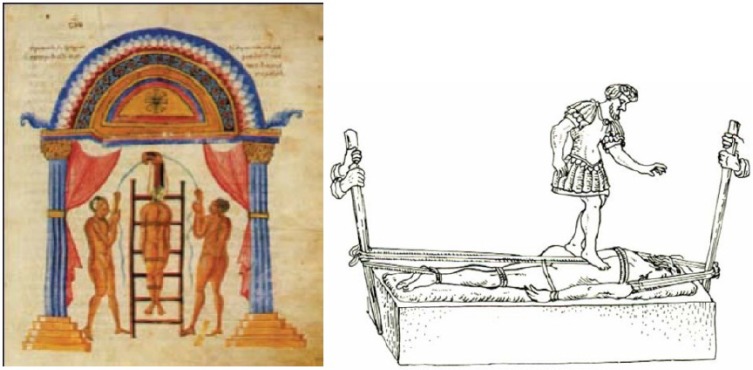
Succussion upon a ladder.

**Fig. (2):**
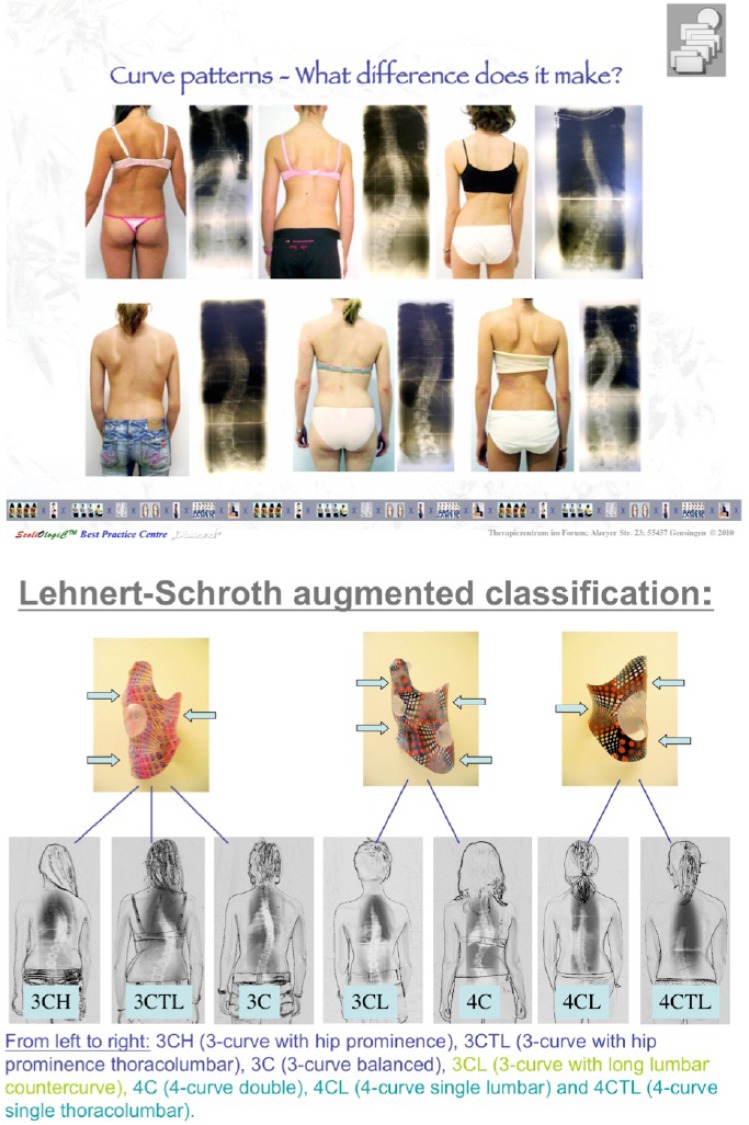
The different curve patterns according to Lehnert-Schroth Augmented Classification. These curve patterns determine the types of scoliosis specific exercises and braces prescribed. From left to right, the curve patterns are respectively 3CH (thoracic curve with hip protruding contralateral to the side of thoracic convexity), 3CTL (thoracic curve with apex at T12 and hip protruding to the other side), 3C(a balanced thoracic curve with very little or no coronal imbalance), 3CL (a thoracic curve with a long lumbar counter curve), 4C (a double major curve, with little pelvis shift), 4CL (lumbar curve with pelvis shifting to the contralateral side) and 4CTL (thoracolumbar curve with apex at L1 and pelvis shifting to the contralateral side).

The term “Scoliosis”, is, however usually attributed to Hippocrates. He recommended “succussion” upon a ladder (Figs. **1a**, **b**, **2**), for cases in which the hump was close to the neck. The patient was bound to a padded ladder, hoisted while still on the ladder to a high tower and extended by manual traction at either end.

It is clear, however, that Hippocrates did not distinguish between antero-posterior and lateral deformities, or to “gibbosities” due to different pathogenesis. Treatment in classical Greek times was a mixture of gymnastics, faith healing, spa treatments, and applied psychology [2]. In the Roman era, the knowledge of mechanics was utilized in the treatment of deformities using principles which are still relevant today. Galen in the 2nd century A.D. advocated direct pressure and traction as well as lever pressure and traction. The Asclepion at Pergamun where Galen originally worked, was a combination spa and rehabilitation centre [1]. The Arabic cultures also contributed to the medical tradition and it is believed that Mohammed al Gafequi of Cordoba (1265) advocated spinal fusion using fish bones [3]. It was not however, till the sixteenth century that Ambrose Paré described the deformity that we recognize today.

Shortly after, Hildanus (1646) illustrated a scoliotic spine, but it was only in the 18th century that a fuller understanding of spinal deformities was achieved. Two persons of note were Andry [1] who wrote the textbook of orthopaedics and further defined and postulated on the pathogenesis of scoliosis, and Robert Chessher of Leicestershire (1751) who treated spinal deformities by first relaxing contracted muscles with fomentations, friction and machinery and then applying splints. This principle is similar to the conservative treatment that is used today; muscles are relaxed or stretched to decrease spinal curvature and the spine is then held in this corrected position by means of isometric exercise, plaster jackets or braces. While the actual definition and scope of spinal deformities continue to evolve, the term itself includes spinal conditions such as idiopathic scoliosis, congenital scoliosis, Scheuermann's kyphosis spondylolisthesis, as well as numerous other spinal pathologies [4].

## 4 WHAT IS ADOLESCENT IDIOPATHIC SCOLIOSIS?

The definition of adolescent idiopathic scoliosis (AIS) put forward by the Scoliosis Research Society is “a lateral curvature of the spine presenting at or about the onset of puberty and before maturity with no associated musculoskeletal condition” [5]. This definition however, gives no indication of the three-dimensional nature of the deformity, as only in a small number of non-rotated scoliosis is the deformity only in one plane. The deformity is more precisely a lordotic lateral rotatory lesion [6], with the lateral component of the deformity being in the coronal plane, the rotational component in the transverse plane and the lordotic component in the sagittal plane.

## 5 TYPES OF SCOLIOSIS

Scoliosis can affect people at different points in their lives. There are many different causes of scoliosis. Over 80% of them are idiopathic with causes unidentified [1].

## 6 PREVALENCE OF SCOLIOSIS IN ADOLESCENTS

Data regarding the prevalence of AIS varies widely. Leatherman and Dickson [6] stated that as many as 15% of 10-14 year old’s screened for scoliosis demonstrated some degree of curvature. Other studies made the issue more complex by dividing prevalence into prevalence above 10° and prevalence above 20° Cobb angle. In Montreal, Canada, a screening programme of 15,000 young adolescents reported that 1.6% demonstrated a curvature greater than 10^o^. Smyrnis *et al.* [7] examined 63,000 children in Athens and found 2.7% to have a curve of 10^o^ or more, while Willner and Uden [8] in Sweden obtained a prevalence of 1.9% in curves greater than 10^o^. They [8] also reported that the prevalence differed significantly between the sexes. Girls had a prevalence of 3.2% as compared to boys with a prevalence of only 0.6%. In other words, female to male ratio was 5.3:1.

## 7 CURVE PROGRESSION FOR AIS

The potential for curve progression is related to several factors; amongst these, the patient's gender, age, Risser sign, curve magnitude, maturity, rate of growth and growth potential at presentation. Dickson and co-authors [9] demonstrated that when curves of 10^o^ and above were considered, the female to male ratio was 1.6:1. This value increased to 12:1 when curves greater than 20^o^ were considered. Female to male ratios for treatment were nearly as high with a reported ratio of 8-10:1 [10]. Lonstein and Carlson [11] demonstrated that when curves of all magnitudes are combined, there is a negative correlation of age with the percentage incidence of progression. In other words - the younger the child, the greater is the likelihood of progression. The same negative correlation was shown with the Risser sign which is graded based on the extent of excursion of the iliac apophysis as it proceeds from the lateral to the medial side where it fuses with the iliac crest. The greater the maturity of the child, the greater is the Risser sign. A low Risser sign indicates a greater potential for growth [11].

Curve magnitude, however, has been found to have a positive correlation with the incidence of progression. Thus, the greater the magnitude of the curve at presentation, the greater is the potential for progression. Other factors taken into consideration when determining progression risk are the changes in secondary sexual characteristics that take place during the growth spurt, as well as the type of curve pattern [12].

Authors have reported different curves patterns that are found to be more progressive. Clarisse [13] and Fustier [14] reported that double curves progressed most in their studies with an incidence of 67% and 75% respectively. Conversely, Bunnell [15] and Lonstein [11] reported that thoracic curves were the most progressive. All authors, however, demonstrated that lumbar curves progressed least. Other parameters of prognostic value include apical vertebral rotation [16, 17] and the rib vertebra angle [18]. When assessing the potential for curve progression, no one factor is taken in isolation, but all factors should be taken into account in attempting to predict the likelihood of curves to progress, before deciding on the best possible treatment.

## 8 MORBIDITY AND MORTALITY IN SCOLIOSIS

Morbidity and mortality in idiopathic scoliosis are directly related to the age of onset of the deformity. Early-onset infantile idiopathic scoliosis which develops from birth to three years of age has the worst prognosis. Increased mortality in this group is largely due to cardiopulmonary compromise. If the deformity is large at the time of development of the pulmonary parenchyma which occurs at about eight years of age, cardiopulmonary problems in later life can be expected [9].

Pulmonary parenchyma development has already occurred in the adolescent group. Thus adolescents who develop spinal deformity after the age of eleven or twelve do not suffer from cardiopulmonary problems in later life. Several long term studies on adolescent idiopathic scoliosis have clearly demonstrated that pulmonary function remains normal even if the curve magnitude is greater than 100^o^ [19, 20]. Branthwaite [21] confirmed these results in an investigation on 800 idiopathic scoliotic patients. Disabling dyspnoea and cardiorespiratory problems were associated with scoliosis of the early onset variety. Only one out of twenty-eight AIS patients who had not undergone surgery developed dyspnoea later in life which could be attributed solely to the spinal deformity.

## 9 UNTREATED ADOLESCENT IDIOPATHIC SCOLIOSIS 

Untreated symptomatic or syndromic scoliosis as well as early onset scoliosis (EOS) can sometimes cause severe health problems and higher mortality. However, adolescent idiopathic scoliosis (AIS), which is the most common form of idiopathic scoliosis, is relatively benign. It does not generally lead to severe health problems or early death [9]. Weinstein *et al*. [22] in a 50-year follow-up of untreated AIS patients has shown that this population functions well. There were no more health problems in this group when compared to patients without scoliosis, other than a slight increase in the prevalence of back pain and cosmetic concerns.

## 10 EVIDENCE OF TREATMENT EFFECTIVENESS IN ADOLESCENTS IDIOPATHIC SCOLIOSIS

As mentioned previously, the indications for treatment largely rely on the Cobb angle, the angle of curvature as measured on an X-ray of the spine in the frontal plane. Historically, the treatment of scoliosis consists of scoliosis-specific exercises (SSE) (15 - 25° Cobb), orthotic treatment (20 - 40° Cobb) and spinal fusion surgery (> 40 - 50° Cobb) [1].

Physiotherapeutic Scoliosis-Specific Exercises (PSSE)

The use of the word ‘specific’ within the phrase PSSE has been widely used to describe exercises that are designed for the treatment of patients with scoliosis [23-26]. However there are clear differences within the different approaches or schools that identify what makes them ‘specific’ compared to general back exercises that can be designed by any professional, in any clinic for the scoliosis patient. Some of the differences are outlined in this section in the hope that this will help to clarify these differences and similarities to assist all involved: the patient and clinician as well as the researcher, in the management of this condition.

Physiotherapeutic Scoliosis-Specific Exercises (PSSE) has been outlined in more depth in previous papers, outlining the different approaches [25-27]. These specialised scoliosis exercises and rehabilitation techniques are widely used and recognised in several central European countries. These include: The Schroth method - German ‘Original Schroth` and in recent years ‘Schroth Best Practice’; the BSPTS (Barcelona Scoliosis Physical Therapy School); SEAS approach (Scientific Exercise Approach to Scoliosis); the Dobomed method; FITS (Functional Individual Therapy of Scoliosis) ; the Lyon method and Min Mehta’s ‘Side-shift’ exercises [28]. Studies published within the last 25 years, written from the conservative scientific community are discussed later in this paper and clearly demonstrate that a number of these approaches are supported by research evidence and can significantly improve clinical outcomes for patients.

What exactly makes them ‘specific’? – Previously the term PSSE was coined to establish that a number of back exercises used specifically in the treatment of scoliosis have similar and distinguishing features. These include:

- Exercises that are individually adapted to a patient’s curve site, curve magnitude and characteristics;
- Performed with the therapeutic aim of reducing the deformity and preventing its progression;
- Aim to stabilize the improvements achieved with the ultimate goal of limiting the need for corrective bracing or the necessity of surgery [25].

All of the approaches above meet these criteria.

Pattern Specific Scoliosis Rehabilitation (PSSR)

In this specific type of PSSE approach clear strategic principles are used with specific patterns of curvature. Mehta’s approach also follows a basic pattern [28] with side-shift exercises and bracing, which was unique for the time and place in the UK, but is limited to a two dimensional curve pattern. The three dimensional pattern-specific approach of Schroth uses the original concepts of Katharina Schroth and the expanded Schroth classifications: 3C/B, 4C/B [29, 30]. These classifications have been further expanded for German bracing by Weiss [30, 31], grandson of Katharina Schroth, to; 3CH, 3CTL, 3C, 3CL, 4C, 4CL and 4CTL in the Lehnert-Schroth classification [31], a pattern also used in German design bracing. Some of these main original principles and patterns were further adapted by Rigo in the Barcelona Scoliosis Physical Therapy School exercise protocol by adding radiographic criteria [32, 33], a pattern also adapted in Spanish design bracing.

A number of pattern specific approaches continue to be simplified for the adolescent idiopathic patients into 4-5 exercises known as ‘Schroth Best Practice’ [31]. These exercises are based on and incorporate the original Schroth principles yet each protocol is designed to challenge the patient’s neuro-dynamics by focusing on upright exercises known as 3D made easy which combine ADL and Schroth breathing for de-rotation and deflexion of the spine and ribcage. This program is also used alongside corrective bracing.

The original German Lehnert-Schroth exercises [29-31] continue to be used mainly for adult patients with spinal deformities. These are patients who are not generally braced (unless pain is reported in relation to posture), present with stiffer more kyphotic curves with greater magnitude and are less likely to be offered surgical procedures; or adult patients not requiring or weaned from bracing.

PSSR (Pattern-Specific Scoliosis Rehabilitation) is used to distinguish this pattern-specific method from other approaches. As already outlined above, these exercises use a pattern classification to simplify exercise prescription. These exercises and postures can easily be adapted to curves of all severities including the younger patient with milder curves and also to the patient with more skeletally mature larger curves. The goal of PSSR instruction is to eventually allow the patient to become independent from the therapist and incorporate what is learned into their everyday activities. These exercises also sit within the criteria for Scoliosis Specific Exercises.

In a number of the approaches the exercises themselves are not aimed necessarily at improving the correction as such, but are used within the approach to challenge the patient whilst they maintain a corrected posture, for example; to complete a balancing task in an unstable position whilst maintaining a postural correction (SEAS). The posture is repeated and taught at increasing levels of difficulty, to affect the neuro-dynamics and is also used alongside Italian bracing [25, 26].

The FITS, Dobomed, and Lyon methods all follow the principles of corrective movement and use the individual presentation of the deformity, they provide the corrective position together with the corrective movement within exercises. They support the use of native bracing but do not necessarily outline a specific set of exercises, nor a pattern but are still specific to an individual patients’ scoliosis. These approaches are outlined in more depth in Bettany-Saltikov *et al.* [25, 27] and Berdishevsky *et al.* [26] by the principal practitioners of these approaches.

PSSR and PSSE all include either a series of pattern-specific or scoliosis-specific exercises or corrective movements or postures which differ depending on the presentation or a pattern of curvature, magnitude and characteristics; performed with the therapeutic aim of reducing the deformity and preventing its progression. They aim to stabilise the improvements achieved with the ultimate goal of limiting the need for corrective bracing or the necessity of surgery. These exercises work mechanically by changing the musculature and other soft tissues of the spine, improving (postural) aesthetics, and also work neuro-dynamically by challenging the postural muscle group movement patterns. Corrective postures, like effective braces, work on the unloading and de-torsion of the spine in an attempt at reducing or realigning the bony structural deformity.

However it is important to remember that all these approaches require a high level or specialised scoliosis training, certification and experience by clinicians in order for patients to receive effective personalised instruction best suited to their specific curve type. Training in these different PSSE approaches is necessary to maintain the skills needed to both use and teach them effectively to patients.

The use of exercise for the treatment of Adolescent Idiopathic Scoliosis (AIS) has always been a subject of much controversy in the UK and USA. Whilst it is routinely used in many countries of Europe, the main Health Service within the UK and USA do not differentiate between general physiotherapy and the more established specific approaches. General physiotherapy is understood to consist of more generic exercises, usually consisting of low-impact stretching and strengthening activities. It has more recently been suggested by Schreiber *et al.*, 2016 [34] that differences between the North American and European guidelines may be due to cost, culture, social standards or possibly differing appraisals of the quality of research involving exercises.

The lack of the differentiation together with the lack of understanding of these interventions in the USA, UK and Australia, may be a likely cause for the lack of the routine use of these approaches. A possible reason for the negative beliefs towards PSSE within the clinical community in the United Kingdom is the lack of knowledge within the physiotherapist community and associated orthotic clinical specialists. These approaches are not taught at either undergraduate or post-graduate level within the physiotherapy curriculum in the UK or USA. Most clinicians (physiotherapists and surgeons) in the UK normally do not appreciate the difference between these specialist approaches and general physiotherapy. It is well documented that general non-curve specific physiotherapy interventions have been shown to be ineffective [35]. This negative association and the lack of scientific interest in conservative measures in the UK and USA over many decades re-affirm this stand point. This negative opinion of the Research Committee of the American Orthopaedic Association can be traced back to a paper published 75 years ago [36].

## 11 THE AIMS OF TREATMENT ARE DEPENDENT UPON THE PATIENT PRESENTATION

The overall aim of PSSE is to reduce the progression of the scoliotic deformity. However depending upon the specific age and risk of progression of the individual patient, PSSE also aims to postpone or avoid brace prescription and ultimately surgery. These approaches have also been reported to reduce the incidence of surgery [37]. Physiotherapeutic Scoliosis-specific Exercises can be used in three main clinical scenarios: (i) the sole use of exercise as the primary treatment of AIS for mild curves, (ii) in conjunction with braces for moderate curves, and (iii) during adulthood if the scoliosis curves exceed certain thresholds. In the treatment of patients with mild scoliosis of less than 25° Cobb, intense three-dimensional spine and rib-cage specific exercises are used in order to try and avoid the use of a brace and further treatments [243, 24].

In mild scoliosis cases where exercise is prescribed, PSSE is predominantly used according to the recommendations of the Society on Scoliosis Orthopaedic and Rehabilitation Treatment (SOSORT) group [35]. The key objectives in mild cases of AIS are the stabilization and elongation of the spine combined with the three-dimensional auto-correction of the spine, pelvis and rib-cage; postural control via sensory-motor feedback and mirror monitoring. Therefore, these exercises can help improve patients’ quality of life by keeping the curve and rib hump under control for as long as possible, thus reducing the need for braces and improving the outcomes for the patient. The SOSORT group guidelines [35] recommend that PSSE is either used alone or additionally it can also be used as an add-on to bracing for patients with curves <45˚; firstly to prevent further curve progression at puberty, secondly to prevent or treat respiratory dysfunction, thirdly to prevent or treat spinal pain syndromes, fourthly to improve aesthetics via postural correction, and to reduce the need for surgery, as also outlined by recent Cochrane reviews [23, 24].

The second main clinical scenario for the use of these approaches is in conjunction with brace treatment. In this case, the aims are to reduce the side effects of wearing a brace (muscle weakness, rigidity, flat back) and to improve the efficacy of the brace. They can also be used before a brace is worn in order to reduce spinal stiffness and improve mobility, thus helping to achieve a better correction [38].

Finally, the third possible clinical scenario is during adulthood. If scoliosis exceeds certain thresholds, significant problems such as back pain, breathing dysfunction, contractures and progressive deformity can develop. These impairments and subsequent disability can be addressed through PSSE [39].

## 12 BENEFITS OF PSSE OVER BRACING AND SURGERY

In comparison to PSSE, several studies have also shown that bracing tends to reduce the quality of life of young patients [40] and is not indicated unless the Cobb angle has exceeded specific parameters [35]. Schreiber *et al.* have stated that when reviewing early studies on bracing stretching back over decades, braces have been shown to elicit negative side-effects in patients such as stress, fear of injury, discomfort and limitation in activities [34].

The body of most recent research and reviews, amongst other papers [41-44] of exercise interventions, and the common theme in all 4 of the most recent RCT’s [34, 45-47] are activities of daily living (ADL) and PSSE (they are scoliosis-specific according to the pattern of scoliosis). They are the most effective exercise and physiotherapy treatment.

With regards to evidence for bracing, a recent review in 2016 describes the current evidence [48]. One meta-analysis [49], one prospective controlled trial [50], one randomized controlled trial [51], and one multi-centre prospective controlled trial [52] were found supporting brace treatment. There are also long-term cohorts studies published in the literature supporting the Boston and Cheneau brace treatment [53-55].

In addition, Landauer and colleagues have concluded that compliance (the amount of time the brace is worn by the patient) and in-brace correction (this refers to the initial curve correction achieved when the brace is first worn by the patient) determine the outcomes of brace treatment [55, 56]. The success rates after wearing the Boston brace have been reported to be about 70% [52] and with the standard Cheneau brace the success rate has been reported to be over 90% [54, 57]. However, the same definition of success has not been applied to all brace studies.

Usually, the standard definition of success is considered to be “no progression” if the curve has progressed by less than 6° Cobb angle or it is defined as an “improvement of 6° Cobb angle or more” [52, 57]. In the RCT [51] by Weinstein *et al.* the rate of success was defined as the prevention of curve progression to more than 50°, while in the Italian paper on the Cheneau brace [54], the rate of success was defined as an improvement to 5° or more, or no change within the limits of ± 4°. All these different definitions unfortunately add confusion as patients, clinicians and researchers are unable to interpret what the real scientific results of these papers are and consequently are unable to judge the “real” effectiveness of the brace.

More modern attempts at bracing, such as the German Gensingen brace have reduced the amount of plastic and improved the corrective effects. These changes have improved compliance and reduced the amount of time that a brace needs to be worn throughout growth, therefore reducing the impact on the patient [48, 58-62] whilst providing the best outcome.

Surgery is clearly more invasive, carries further risks and possibly further pain and does not always provide favorable outcomes [23, 24]. Exercises provide a non-invasive means of control, although it is possible that they too have (to some degree) an impact on the quality of the patients’ life. This suggests that the shorter and more effective programs should be researched further.

## 13 PLASTER CASTS AND BRACES FOR AIS: BRIEF HISTORICAL CONTEXT AND CURRENT TREATMENT

### 13.1 Brief Historical Context

Plaster body casts have been used since 1875 when Louis Sayer first described them but it was not until 1952 that plaster casts were used effectively. Initially the Turnbuckle cast was used in the 1920's as an adjunct to the pre-operative and post-operative treatment of scoliosis [63]. Subsequently in the 1950's Risser modified this cast and developed the localizer cast which was used in some centres for the non-operative treatment of scoliosis. Here correction of the curve occurs with the patient's trunk in alignment with longitudinal traction applied by head and pelvic traction. Specific pads are then applied postero-laterally to localized areas to apply pressure through the ribs or transverse processes to the apex of each curve [64]. Another plaster cast technique in widespread use up to 20 years ago is the E.D.F. (Elongation, Derotation, Flexion) technique which was introduced in the 1960's by Yves Cotrel and George Morel [65]. The principle here is to apply the necessary vertebral elongation, as well as thoracic derotation to reduce the rib hump, combined with lateral flexion of the spine to obtain a reduction in spinal curvature.

A major milestone in the non-operative treatment of scoliosis was the introduction of the brace. It was first introduced by Blount *et al.* in 1945 and initially replaced the distraction cast as a corrective device preceding and following spinal fusion. Blount later introduced the Milwaukee brace which is used in some centres up to the present day. Mostly however the Milwaukee brace has been replaced by the more aesthetically pleasing Boston brace [66]. Short term results of bracing varied widely with some authors claiming up to 50% improvement, while others claimed no change in curvature in the short term assessment [66-68]. Long term follow-ups are even more disappointing with a large majority of studies demonstrating the same Cobb angle after four years of brace treatment as before treatment [69].

### 13.2 Current Treatment

Since the 70`s and for the last 40 years or so the effectiveness of brace treatment has been unclear and controversial. Emans *et al.* (1986) reported a 50% curve correction with brace usage [70]. This result however significantly overestimated brace effectiveness as X-rays were taken while the patients were actually wearing the brace. Off-brace X-rays following treatment were not included in the study [70]. Other authors [68, 71, 72] reported that the optimal result of bracing resulted in curves that measured exactly the same at the end of treatment as at the beginning.

However a fairly recent seminal RCT conducted by Weinstein *et al.* in 2013, demonstrated to the research community that bracing was definitely effective in the management of adolescents with idiopathic scoliosis patients [51].

## 14 SURGERY FOR ADOLESCENTS WITH IDIOPATHIC SCOLIOSIS

### 14.1 Brief History and Current Evidence of Treatment

Historically the modern era in the operative treatment of spinal deformities began in the last century in 1911 when Hibbs performed the first spinal operation. This procedure was initially performed for spinal tuberculosis but fusions for scoliosis were started in 1914. It was not until 1924 however that he reported his results with cast correction and spinal fusion. Joseph Risser his pupil added a more versatile and effective hinged plaster and in 1952 developed the localizer cast. From the 1930's to the 1960's the treatment of scoliosis was greatly advanced at the hospital for special surgery by John Cobb. Up to this day many surgeons use the Cobb method for assessing the degree of spinal curvature from x-rays [73].

In the operative treatment of scoliosis, Paul Harrington was the first to introduce instrumentation to spinal fusions in 1953. Initially these operations were performed on polio patients who had a secondary scoliosis. One of his main objectives at this time was to observe the reaction of the spine to the metal implant and the holding and correcting ability of the instruments. The instrumentation consists of a racheted rod with hooks to obtain and maintain distraction. The distraction rod is anchored at both proximal and distal ends of the scoliotic curve with hooks anchoring in a sublaminar manner. The instrumentation has provided a relatively safe way of gaining maximal correction and was the most frequently used procedure at the time.

**Fig. (3):**
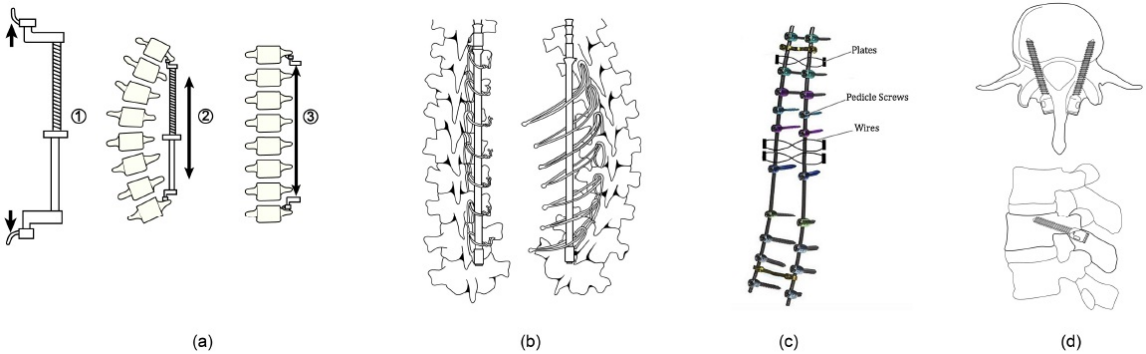
The posterior surgical approach. (a) The Harrington rod with the proximal and distal anchors or hooks. It provides distraction to the scoliotic spine on the side of concavity to reduce the curve. (d) The Luque wiring improves the stability of the Harrington instrumentation. (c) Cotrel-Dubosset instrumentation consists of two rods which are anchored by wiring and segmental screws. (d) The pedicle screws that are widely used today. They are anchored on the base of the pedicle which is always in the lateral half of the superior facet.

In 1970, Eduardo Luque introduced segmental wiring; wires were passed beneath the lamina and around the Harrington rod to strengthen fixation to the spine. Drummond modified the anchor of the wire to the base of the spinous process and termed this the Wisconsin wiring technique [74]. The next improvement was the Cotrel-Dubosset approach which employs two rods, which allow segmental fixation through lamina hooks and/or conical pedicle screws. The system optimizes the de-rotation of the rods and permits correction of the sagittal profile. The combination of rods, hooks and pedicle screws enables distraction, compression, translation, lordosing and kyphotizing of the spine and allows correction of curves in coronal and sagittal planes. The next evolution in the posterior approach is the increased use of pedicle screws (Fig. **3**) in the thoracic and lumbar curves. The primary advantages of pedicle screws over the hooks and sublaminar wires include improved pullout strength and three-column fixation. This improves the stability of the construct and improves control in three planes [74].

Anterior instrumentation with fusion was first proposed by Dwyer and Schafer in 1974, based on the theories developed by Klaus Zielke for thoracolumbar and lumbar curves. The technique was more commonly used in 1980, following the technique refinement by Dr. Jürgen Harms [75]. Anterior instrumentation can be used for discectomy and to improve curve correction after posterior instrumentation. The excision of the growth plate anteriorly will prevent further growth anteriorly and possible deterioration of the curve after surgery [76].

In the past years, there has been a reduction in anterior approach surgery. Large open thoracotomy may compromise pulmonary function. Also, thoracoscopic discectomy was difficult. The strong corrective forces and high fusion rates of all pedicle screw constructs [75] may also contribute to the reduction in anterior approach surgeries.

**Fig. (4):**
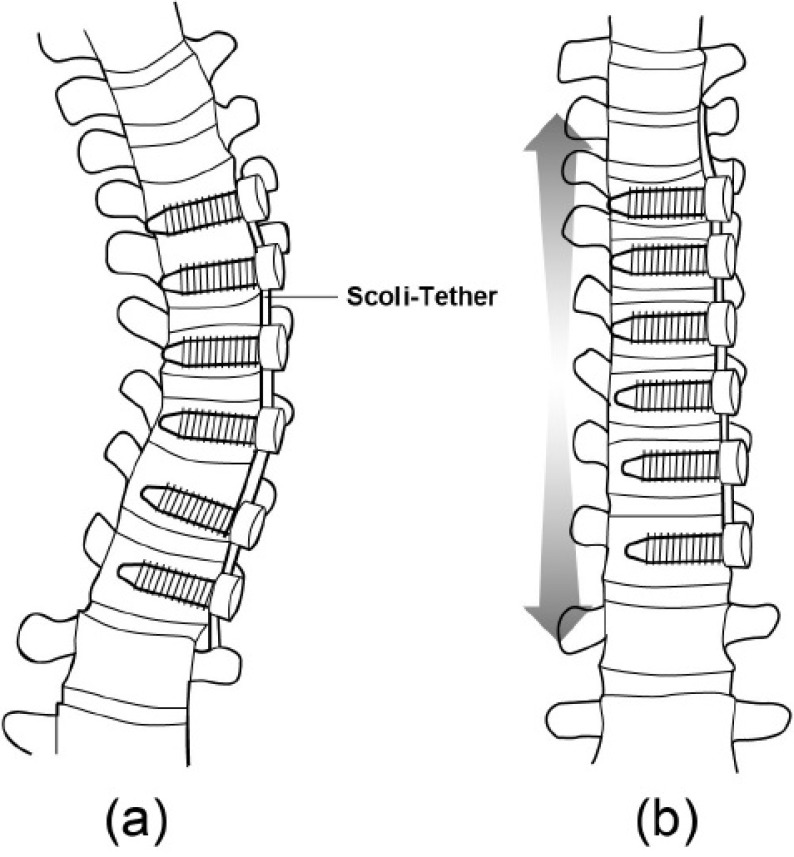
The anterior vertebral tethering approach. This involves anchoring a tether by screws which are advanced from the convexity of the curve toward concavity across the anterolateral aspect of vertebral body, with the objective of modulating the growth of vertebral body on the side of convexity.

Vertebral body stapling and vertebral body tethering are the more recent surgical techniques, which aim at modulating vertebral growth on the side of the curve convexity (Fig. **4**). Vertebral body stapling involves stapling across the physeal end plates and discs of adjacent vertebra to modulate the vertebral growth plate. It is indicated in patients who have thoracic curves <35^o^ or lumbar curves <45^o^ and who are non compliant with brace wear. Vertebral body tethering is indicated in immature patients with thoracic curve between 30 to 65^o^ [75, 76], and bend film X-rays showing a reduction of the curve by 50% [77]. Yet, it is of note that the indications fall within the treatment indications of conservative treatment of AIS [78]. Also, the re-operation rate exceeded 50% within two years of surgery [79].

There is worldwide general agreement that patients with curves in excess of 45^o^ are considered candidates for surgery. The selection of instrumentation and operative approach is dependent on the curve location, magnitude, curve flexibility and sagittal alignment [80]. In large rigid curve, the anterior-posterior approach is generally adopted.

## 15 COMPARISON OF SURGICAL TO NON-SURGICAL INTERVENTIONS IN AIS PATIENTS

### 15.1 Health-Related Quality of Life (HRQoL)

Medium to long term studies have shown that AIS patients treated by bracing and surgery had little differences in quality of life, as measured by HRQoL or SF-36 [81, 82]. Andersen *et al.* (2006) reported the outcome of 181 patients, 9.7 years after being treated by brace or surgery [81]. They found that patients generally had a high level of Activities of Daily Living (ADL). No statistically significant differences between Braced Treated (BT) and Surgically Treated (ST) patients were found for any of the SF-36 variables. Compared with age-matched controls, the SF-36 scores were lower in the AIS patients. Brace related questions revealed a significant impact of the disease and the treatment on the patients’ lives. The study concluded that patients had moderately reduced perceived health status and ADL, and increased pain with the ST patients generally at a better level than the BT [81].

A long-term study, using Harrington rod had similar findings [82]. In a study which evaluated the health-related quality of life in patients with adolescent idiopathic scoliosis at least 20 years after treatment with brace or surgery, the authors found no differences in terms of socio-demographic data between the groups [82]. Patients treated for adolescent idiopathic scoliosis were found to have approximately the same HRQL as the general population. Only a minority of patients (4%) had a severely decreased psychological well-being, and a few (1.5%) were severely physically disabled due to their back pain [82]. Recently, Ward *et al.* (2016) reported that there were no meaningful clinically significant differences in SRS-22r scores at an average 8 year follow-up between AIS patients with curves ≥ 40^o^ treated with or without surgery [83].

### 15.2 Radiologic Findings and Curve Progression 22 Years After Treatment

Danielsson and Nachemson (2001) reported the long-term outcome of a follow-up investigation of a consecutive series of AIS patients treated between 1968 and 1977 [84]. In this series, 156 patients underwent surgery with distraction and fusion using Harrington rods, and 127 were treated with brace. The mean follow up times for surgically treated patients was 23 years and for braced treated patients was 22 years. Results showed that both groups of patients had more degenerative disc changes than the normal control participants. No significant differences, however, were found between the scoliosis groups [84]. Although more than 20 years had passed since completion of the treatment, most of the curves did not increase. The surgical complication rate was low and degenerative disc changes were more common in both patient groups than in the control group [84].

### 15.3 Childbearing, Curve Progression, and Sexual Function

Danielsson and Nachemson (2001) also showed that surgical or braced treatments of AIS patients did not impact childbearing, curve progression and sexual function significantly [85]. Overall patients appeared to function well with regard to marital status and number of children. The scoliotic curve did not seem to increase as a result of childbearing. Minor problems occurred during pregnancy and delivery. Some patients, however, experienced a slight negative effect in their sexual life [85].

### 15.4 Back Pain and Function 22 Years After Brace and Surgical Treatment

The same authors Danielsson and Nachemson (2003) also reported on back pain and function 22 years after brace and surgical treatment on the same group of patients as above [86]. Brace treated AIS patients had a mean curve progression of 7.9^o^ [86]. They had more degenerative disc changes; yet they had minimal pain and no dysfunction when compared with normal controls. The mean end result was similar to that of the surgically treated group, except that the pain of the braced treated patients had a more affective (emotional) component.

### 15.5 Spinal Range of Motion, Muscle Endurance, Back Pain and Function

The same consecutive series of AIS patient were reported by Danielsson and Nachemson 2003 to have lumbar motion and muscle endurance significantly reduced when compared with healthy controls [87]. For surgically treated patients, better lumbar muscle endurance or lumbar mobility correlated with a better physical function. Brace treated patients with reduced lumbar mobility experienced low back pain more often than the controls [87].

### 15.6 Pulmonary Function

Pehrsson *et al.* (2001) studied the pulmonary function of patients 25 years after surgery or initiation of brace treatment [88]. They found that the vital capacity of both groups of patients increased. The mean increase for the surgical group was 10.8% and that for the brace treated group was 12.3%. No significant correlations were found between vital capacity and Cobb angle pre-treatment, post-surgery, nor with the difference between the Cobb angles before and after surgery. Smoking and curve size were not found to be risk factors for reduced pulmonary function [88].

### 15.7 Summary of Treatment of Adolescent Idiopathic Scoliosis

Long-term follow up studies have shown that AIS is relatively benign. Patients do not generally have more health issues than those without scoliosis, apart from a slight increased prevalence of low back pain and cosmetic issues. RCTs have shown that both PSSEs and scoliosis orthoses are effective in stabilizing the curves in AIS patients. In some patients, the curves improve. Surgery which is generally indicated for curves in excess of 50^o^ is not supported by a high level of evidence. In light of the relatively benign nature of AIS, it is suggested that the indication of the curve range treated conservatively can be increased to beyond to what is currently presumed [35, 78].

### 15.8 Adult Scoliosis

Adult scoliosis can be divided into two main types [89]. It is known that some of the curves that occur in a growing child may increase as he or she progresses through adulthood. It has been proposed that curves of ≥50^o^ after skeletal maturity may worsen by an average of 1^o^ per year whereas curves of less than 30^o^ rarely worsen [90, 91]. An examination of longitudinal studies involving adolescent idiopathic scoliosis [92] has suggested a much more variable rate and natural history of curve progression. This type of deformity is sometimes referred to as Adult Scoliosis of Adolescent Onset [ASAO].

The second category of scoliosis which occurs in adults is referred to as degenerative or “de novo” scoliosis. This category of spinal deformity starts after the age 40 and is thought to be the result of degeneration of the spine, with changes in alignment due to degeneration of both discs and facet joints. De novo curves may progress slowly or more rapidly, particularly in the presence of osteoporosis and subsequent vertebral collapse [93].

### 15.9 Prevalence of Scoliosis in Adults

Studies from the USA have estimated that the incidence of scoliosis and other types of spinal deformity in the adult population (mean age 70.5 years) are as high as 68% [94]. Earlier studies have given figures as low as 32% [95]. The study quoting the highest figure found no significant correlation between the incidence of scoliosis and pain. A more recent study from South Korea [96] with an inclusive definition of Cobb angle ≥ 10^o^ found the prevalence to be 35.5% in older people. The prevalence of kyphosis in the adult population, although anecdotally more prevalent [97, 98] than other deformities is much less documented. However figures of between 20 and 40% of the elderly adult population have been quoted [99]. If we accept these estimates as comparable to the UK population, the numbers of patients presenting with adult scoliosis is due to increase. During the period 1985-2010, the number of people aged 65 and over in the United Kingdom increased by 20 per cent to 10.3 million. By 2010, 17 per cent of the U.K population was aged 65 and over. The number of people aged 85 and over more than doubled over the same period to reach 1.4 million and the percentage aged under 16 years fell from 21 to 19 per cent [100]. It is predicted the population aging will continue for the next few decades [100]. By the year 2035, the number of people aged 85 and over is projected to be almost 2.5 times larger than in 2010, reaching 3.5 million individuals and accounting for 5 per cent of the total U.K. population. The population aged 65 and above will account for 23 per cent of the total population by 2035. The proportion of the population aged between 16 and 64 years is due to fall from 65 per cent to 59 per cent [100]. A recent study has indicated increased morbidity and mortality rates associated with spinal surgery in more elderly patients [101]. In this study running from 2005 to 2008, the mortality and complication rates of the 3475 patients undergoing spinal surgery in that period were registered in a database. The average age of these patients was 55.5 years (range, 16 to 90 years) and 54% of this cohort was men. Ten patients (0.3%) died after surgery, and there were 407 complications in 263 patients (7.6%). Increased patient age and infected wounds were identified as independent predictors of mortality [101].

### 15.10 Curve Progression in the Adult Population

Adult spinal deformity is a common disorder that can have a significant and measurable impact on an individual’s health-related quality of life. There are significant differences between the adult and the adolescent with spinal deformity and these include patterns of deformity, degenerative components, and the natural history of deformity progression, clinical symptoms, and initial presentation [91, 102]. The goals of operative and non-operative care, and surgical strategies for achieving these goals of care, can therefore differ significantly between adult and adolescent patient groups. Deformity in the adult spine is often characterized by associated degenerative changes, including spinal stenosis, spondylolisthesis, subluxation, lumbar hypo-lordosis, and stiffness within the deformity [91, 103]. Two of the most significant presentations of spinal deformity within the adult population are scoliosis and kyphosis.

### 15.11 Conservative Management in Adults

Not every adult with scoliosis or kyphosis requires surgical treatment. In fact, the vast majority of adults with deformity do not have any disabling symptoms and their condition can be managed with simple measures such as periodic observation, non-prescription analgesics and exercise. It has been suggested that adult scoliosis patients suffer a similar level of pain to those of a similar age with nonspecific lower back pain. Exercise and physiotherapy are aimed at strengthening the core muscles of the abdomen and back and improving flexibility [104]. Should these approaches fail, steroid or local anaesthetic injections in the muscle, joints, or spinal canal may be considered. For persistent neurological leg pain and other symptoms due to arthritic change, injections such as epidurals, nerve blocks or facet injections may provide some temporary relief. The goal of such injections is both diagnostic and therapeutic [105]. Patients can track their response to the various injections in order to help define locus of their pain. Review of pain management within an outpatient based pain management team can subsequently lead to prescription of stronger analgesia [105]. A major drawback of using stronger pain medications is that they can have a sedative effect, cause unwanted long term side effects and be addictive [105]. Such pain relief therefore has to be used with caution.

If the patient has osteoporosis, this must be addressed as well. Combinations of calcium, vitamin D, and oestrogen have been used [106]. Calcitonin has been used in some cases to inhibit the breakdown of the minerals in bone, and fluoride has also been used in an attempt to increase bone mass. A recent innovation is the use of drugs in the bisphosphonate family to help maintain and possibly increase bone mass [106]. These medications can help decrease pain but cannot correct wedged bone or spinal deformity. If these conservative measures do not help, surgery may be necessary to control pain and improve deformity and /or decompress affected nerve roots, though it has to be noted that surgery in adult with spinal deformities is associated with a high rate of complications [101]. Sciubba *et al.* (2015) reported an overall mean complication rate of 55% for surgery of adult spinal deformity [107].

### 15.12 Orthotic Treatment of Spinal Deformity in Adults

Early orthotic treatment (use of bracing) is increasingly cited as a non-invasive and cheaper treatment method for many musculoskeletal problems [108]. The online catalogue of NHS supply chain lists 795 orthoses to immobilise or protect the spine [109]. Although this list included orthoses not designed specifically to treat adult spinal deformity and includes duplicate products and designs from different contractors, the range of orthoses available is clearly large. The primary aim of orthotic management of spinal deformity is to stop curvature progression. Improvement of pulmonary function (vital capacity) and treatment of pain are also considered as major aims of the treatment [78]. In most instances this treatment is applied via a Thoracolumbosacral Orthosis (TLSO). An orthosis has been defined by International Organization for Standardization (ISO) as “an externally applied device used to modify the structural and functional characteristics of the neuromuscular and skeletal system” [110]. A thoracolumbosacral orthosis (TLSO) is an orthosis that encompasses in whole or part the thoracic, lumbar and sacral portions of the spinal column. TLSOs are prescribed to control motion, correct deformity and/or compensate for weakness [111].

As has been mentioned above, many types of these devices have been developed either as “off the shelf” orthoses which are modified from a basic, sized kit [112-114], or as a bespoke device, manufactured from measurements, plaster of Paris casts [115] or a scan of the wearer’s body [61]. Although guidelines exist on the use of spinal bracing to treat adolescent idiopathic scoliosis, no such advice exists for orthotic treatment with adult onset scoliosis other than for adults with a Cobb angle greater than 30^o^ where the advice given was “outpatient physical therapy, Scoliosis Intensive Rehabilitation program [SIR], where available” [78].

The long term use of spinal bracing has been discouraged as they are said to weaken the core muscles. In one study [104] looking at paediatric patients with neuromuscular scoliosis, the effects of wearing a thermoplastic Thoraco-Lumbar-Sacral Orthosis (TLSO) on respiratory function were investigated. Wearing the devices reduced the forced vital capacity by 17.56%. Following a regime of physiotherapy, this figure fell to 9.28% [104]. Thus the authors argued that a tailored physiotherapy program should be used in combination with a prescription of a TLSO [104].

A similar study [116] looking at the effect of bracing on Total Lung Capacity (TLC) on a population of AIS patients found that although the braces did have the effect of reducing TLC, the degree of this effect decreased as the deformity (measured as Cobb angle) increased [116]. Although many authors have debated the optimal angle to brace AIS [78], this is not the case with adult onset scoliosis. These studies often stated the lack of heterogeneity of study population for the varying results [117] and this will potentially be the same in adult studies. Studies looking at the effect of pubertal growth spurts on curve progression in AIS [118, 119] could be compared to the later demineralisation of bone during the menopause and old age.

A multi-centre study from the United States investigated the use of non-surgical treatments in patients with adult spinal deformity [120]. These patients were divided into high and low symptom groups, which comprised 17.5% and 12.5% of the group of patients respectively. They used spinal bracing as part of the treatment for pain and deformity [120]. A later paper investigating the costs and benefits of using conservative treatment for adults with scoliosis [121] reiterated the earlier reports’ findings of poor evidence to support the use of conservative management. Although this study mentioned bracing in its executive summary, this form of treatment was not discussed in terms of its therapeutic effect, and was not included in the tabulated results. The Scoliosis Association (UK) in its factsheet discusses the ability to treat the pain associated with adult onset scoliosis conservatively, but does not elaborate on possible modes of treatment.

### 15.13 Surgical Treatment

Surgical treatment may be indicated if the curve increases or other associated symptoms worsen. The type of surgical procedure chosen varies depending on the nature and magnitude of the curve. A recent study has suggested the categorisation of patients depending on their degree of spinal stenosis related pain and/or deformity to inform future surgery type [122]. Patients with symptoms of stenosis were treated primarily by decompression, with or without concomitant fusion [122]. Patients with symptomatic or progressive deformity were treated primarily by surgical fusion, with or without decompression [122]. It has to be noted that smoking and some medical conditions can affect post-surgical healing, recovery and re-operative rates [123]. A brace may also be used to stabilise the spine during the post-surgical healing process.

### 15.14 Adolescents with Hyperkyphosis

Little is known even amongst health service professionals about physiotherapy, exercise rehabilitation and brace treatment for patients with hyperkyphosis. Physiotherapy for postural improvement is often recommended, especially in central Europe, focusing on pectoralis and hamstring stretching and trunk extensor strengthening as well as improving function in activities. Combinations of these types of exercises also make up modern and more established approaches to exercise treatment described in the section below.

### 15.15 Scheuermann’s Disease

Scheuermann's disease initially was described as a rigid kyphosis associated with wedged vertebral bodies occurring in late childhood [124]. Scheuermann's disease has been of significant orthopaedic interest in the past, because it sometimes may be painful during its relative acute phase and more importantly, because it may cause significant trunk deformity that can progress. Sorensen subsequently described specific criteria for diagnosis in 1964 [125], namely, that three adjacent vertebrae must be wedged at least 5° each. Others articles have used different criteria as outlined in a recent review [126]. These include increased thoracic kyphosis, disc space narrowing and irregular endplates associated with a single-wedged vertebra, a kyphosis of greater than 45° with two or more wedged vertebrae, or “characteristic” radiographic findings, kyphosis wedging of vertebral bodies, endplate irregularities, Schmorl's nodes [127, 128].

The etiology of lumbar Scheuermann's kyphosis is mostly unknown, but strong associations with repetitive activities involving axial loading of the immature spine favour a mechanical cause [127]. Although the radiographic appearance may be similar, lumbar Scheuermann's kyphosisis is regarded as a different entity than thoracic Scheuermann's kyphosis [126, 129]. Unlike classic thoracic Scheuermann’s kyphosis, the treatment of lumbar Scheuermann's disease was not controversial in 1999 [126, 129], as its course has been regarded as being non progressive and its symptoms have been regarded to resolve with rest, activity modification and time [130-132].

However, this loss of lumbar lordosis, in the lumbar or thoracolumbar areas means that Scheuermann's disease can be one of the predictors of developing chronic low back pain in adulthood; loss of lumbar lordosis correlates well with the incidence of chronic low back pain in adulthood [133, 134]. Sedentary lifestyle contributes to loss of lumbar lordosis as well as scoliosis and thoracolumbar or lumbar kyphosis [130, 135, 136]. It is necessary to recognise the severity of symptoms in patients with back pain, as they increase in a linear fashion with progressive sagittal imbalance. The results of these studies also show that hyperkyphosis is more favourable in the upper thoracic region but very poorly tolerated in the lumbar spine [130, 133, 134]. It has been shown that lumbar re-lordosation stabilizes the spine with respect to lateral deformity [135], so we may assume lumbar de-lordosation or lumbar kyphosis destabilizes the spine and can lead to chronic low back pain. This is the underlying physiological reasoning in established treatment programs.

According to Wenger and Frick [129] the incidence of Scheuermann's disease has been estimated to be at 1 to 8% of the population [125, 134]. The typical presentation is in the late juvenile age period from 8 to 12 years, with the more severe fixed form commonly appearing between age 12 and 16 years. Patients with thoracic roundback, who have classic type I Scheuermann disease, may have pain in the thoracic spine area, but more frequently present because of patient and parental concerns related to trunk deformity (aesthetic symptoms).

Patients with Scheuermann's kyphosis have an angular thoracic kyphosis often with accompanying compensatory lumbar lordosis and increased cervical lordosis. The position of the head is often in forward protrusion (excessive poking-chin posture), and the shoulders are often positioned anteriorly. Forward bending typically accentuates the kyphotic deformity, with a sharply angulated bend noted in the thoracic or thoracolumbar region. The deformity is relatively fixed, remaining flexed even when attempts are made to extend the spine. Tightness of the hamstrings is common, but the neurologic exam is usually otherwise normal [126, 127]. Unfortunately Wenger and Frick [126, 129] do not describe the clinical findings of other curve pattern than thoracic Scheuermann, although the thoracolumbar and lumbar Scheuermann curve patterns are of major importance with respect to chronic low back pain in adulthood [130, 135].

The degree of kyphosis on the lateral film is measured using a modified Cobb method according to Stagnara [137, 138]. In addition to increased measurable round-back on the lateral view, vertebral wedging is used to clarify the diagnosis. Associated findings of scoliosis and spondylolysis can occur with Scheuermann’s kyphosis, but usually are minor and do not alter treatment [126, 129]. The natural history of Scheuermann's disease remains controversial. The condition tends to be symptomatic during the teenage years but often in late teenage life produces less pain [125].

In a long term follow-up study, Sorensen [125] noted pain in the thoracic region in 50% of patients during adolescence, with the number of symptomatic patients decreasing to 25% after skeletal maturity [126, 129]. The pain was described as mild and not incapacitating. Later authors offered a contrasting view of the symptoms of untreated Scheuermann’s disease, with Bradford stating that adults with Scheuermann's kyphosis have a higher incidence of disabling back pain than the normal population [139, 140]. Murray, Weinstein, and Spratt have performed a study designed to describe the natural history of Scheuermann's kyphosis [141. They studied 67 of a group of 118 (57%) patients diagnosed by the Sorenson criteria, using physical examination, trunk strength measurements, radiography, a detailed questionnaire and pulmonary function testing [126, 129]. The patients had an average kyphotic deformity of 71°, and the average follow-up was 32 years; an age-matched comparison group was used as controls. They concluded that patients with Scheuermann's kyphosis may have functional limitations, but these did not result in severe limitations due to pain, or cause major interference with their lives [126, 129]. In another paper, Lowe and Kasten [142] state that adults with more severe deformities (>75°) secondary to untreated Scheuermann's disease can have severe thoracic pain secondary to degenerative spondylosis and can be significantly limited by their disease [142]. The authors concluded, the greater magnitude of the deformity as a possible explanation for the life-altering pain experienced, in contrasted to those reported on by Murray *et al*. [142], although there is no evidence to demonstrate a direct correlation between deformity and pain [126, 129].

The common indications for treatment in Scheuermann's kyphosis are related to pain, progression of deformity and appearance (aesthetic issues). Pain is difficult to measure because of its subjective and temporal nature [126, 129]. Most of the literature on Scheuermann's kyphosis states that pain is either present or absent, and does not provide data on how this was determined or measured [126, 129]. The study by Murray *et al*. [141] is the only single attempt in the literature to objectively assess pain in this patient group. They found no statistically significant difference between the Scheuermann patients and the control group with regard to the extent that pain interfered with their lives, although it is possible that a clinically significant difference might exist as 38% of the Scheuermann patients had severe interference of pain with activities of daily living compared to 21% of control subjects. The kyphotic group did have significantly higher pain intensity readings, and complained more frequently of pain in the thoracic region than the control group.

Tribus has outlined the reasons for treatment of Scheuermann's kyphosis also for cardiopulmonary compromise [143]. Deformity is the most common complaint of patients with Scheuermann disease, and is typically the primary reason younger patients seek medical attention [126, 129]. However, the likelihood of progression of a kyphotic curve of any given degree of severity is currently not known [144]. Studies reporting on the natural history of lumbar and thoracolumbar Scheuermann's disease were not found.

Treatment and initial management of the patient presenting with Scheuermann's kyphosis includes documentation and assessment of the degree of deformity and/or pain, as well as an overall picture of the negative impact of the deformity on the patient's life [126, 129]. Physiotherapy for postural improvement exercises is often recommended, especially in central Europe, focusing on hamstring stretching and trunk and back extensor strengthening as well as improving function in activities [145]. Specialist physiotherapists can also assess whether there is any tendency toward increased hip flexion contracture and may work on associated lumbar lordosis [126, 129]. There are no conclusive studies documenting improvement in kyphosis with exercises [126, 129], although Bradford *et al*. did note some improvement in patients with moderate degrees of deformity [139]. Scheuermann's disease in adults is regarded to be a different entity from that of the teenager for the major manifestation is pain and not aesthetic quality. The patient's occupation is rather sedentary; sport is beneficial. The functional rehabilitation is the initial treatment choice and recourse to surgery or dorso-lumbar braces is rare [146].

According to Pizzutillo [147] effective interventions for adolescents with postural kyphosis include exercises to relieve lower extremity contractures and strengthen abdominal musculature coupled with practiced normal posture in stance and in sitting. Skeletally immature patients with Scheuermann's kyphosis benefit from a similar exercise program but also require the use of a spinal orthosis. Bracing of the spine in patients with Scheuermann's kyphosis can result in a permanent correction of vertebral deformity, unlike bracing in patients with idiopathic scoliosis. The evaluation and assessment of children and adolescents with increased thoracic kyphosis is an important aspect of the decision process used to determine appropriate interventions and takes specialist professionals [147].

### 15.16 Physiotherapy for Hyperkyphosis

Physiotherapy for patients with thoracic kyphosis has been described at length by Lehnert-Schroth [148, 149]. The principle content of the Schroth exercises for scoliosis does not differ greatly to the general exercises described. For example; positional and actively stretching of the anterior pectoralis muscles, addressing the posterior kyphotic hump are the main principles of physical exercises, along with lower extremity muscles and back extensor strength, within the Schroth exercise program. But like the case of scoliosis-specific exercises, these specialist pattern specific sagittal plane exercises for hyperkyphosis, place the patient in the optimal pattern-specific (according to the established Schroth curve pattern) over-corrected optimal position according to gravity and then help to shorten or stretch the affected muscle groups. These exercises are specific to the pattern and although they contain similar principles of general exercises, training in the curve patterns is essential to prescribe these specific Schroth exercises effectively. The lumbar lordosis needs restoration also. Thoracolumbar and lumbar curve patterns have to be addressed differently by specialist physiotherapy. Pattern-specific hyperkyphosis exercises to improve lumbar lordosis have been described at length [149].

These exercises have been defined as ‘physio-logic exercises’ as they were developed to restore a physiologic lumbar lordosis alongside restoring lumbar function and stability. Historically, kyphosis patients were treated with a four-week in-patient rehabilitation program, especially in Germany. There is a lack of evidence for in-patient rehabilitation and in view of its relatively benign nature, the intensity seems unnecessary. As outlined in recent paper [150], there are 4 main exercises, with variants of each that correlate to the curve pattern in an outpatient setting that claim to be as effective.

### 15.17 Brace Treatment for Hyperkyphosis

The few available studies on the efficacy of brace treatment are retrospective, have different inclusion criteria, and do not have control groups. In addition, as noted above, we do not yet have data available to allow us to predict which kyphotic curves are at significant risk of progression [126, 129]. Despite these shortcomings, bracing is widely regarded as being efficacious in the treatment of Scheuermann's kyphosis in the skeletally immature patient [143, 151]. Bracing has been used primarily for the treatment of deformity, with results of treatment focusing on improvement in kyphosis; the results of brace treatment for relieving pain have not yet been published [126, 129]. The overall results of brace treatment seem reproducible [81] and promise a permanent correction of vertebral deformity, unlike bracing in patients with idiopathic scoliosis [147]. According to Lowe [152, 153] brace treatment is almost always successful in patients with kyphosis between 55^o^ and 80^o^ if the diagnosis is made before skeletal maturity. For kyphosis greater than 80^o^ in the thoracic spine or 65^o^ in the thoracolumbar spine, surgery is indicated in symptomatic patients [152]. Surgery improves the deformity significantly in symptomatic adolescents with severe deformity [153]. However, the studies available do not have strong level of evidence supporting surgical treatment [153].

Surgical treatment in adolescents and young adults should be considered if there is documented progression, refractory pain, loss of sagittal balance, or neurologic deficit. Further negative effects of bracing have been reported [154]. A newly designed brace in the treatment of adolescent Scheuermann thoracic kyphosis has been proposed by Riddle *et al.* [155]. However, the authors did not report on in-brace corrections. It was recommended that this brace be worn until skeletal maturity; in this study the time period was determined to be at least 16 months to induce improvement or halt progression of this disease. Flexible curves are a positive predictor of a successful outcome of bracing with the kyphosis brace.

The kyphologic brace leads to in-brace corrections comparable to those of the Milwaukee brace, which have been shown to lead to a beneficial outcome in the long-term [147, 152, 153, 155-159]. There are other curve patterns than thoracic ones. Within bracing and exercises some distinguish between thoracic, thoracolumbar, lumbar hyperkyphosis or Scheuermann desease. These patterns dictate the brace and exercise used. [126. 130, 135]. Treating the pain caused by loss of lordosis is prioritised over cosmesis in this bracing system. In-brace corrections for this rare pattern of Scheuermann disease, however have not been investigated.

As previously reported [126, 129], surgical interventions come with increased risks. Nevertheless recent publications appear to support surgery [159, 161]. As there is a consensus, that surgery is rarely necessary because conservative management is highly effective [126, 129], it is necessary to improve the conservative standards of treatment. To improve the compliance for conservative treatment and therefore developing a bracing design with less materials but promising in-brace correction seems a step towards improving the effects of correction.

There continues to be a lack of evidence about the current approaches of physiotherapy for hyperkyphosis. Although there is little evidence that physiotherapy alone can change the natural history of the disease, understanding the condition according to patterns can assist the prescription of certain exercises for the treatment of thoracic curve patterns [139, 140, 156]. Lumbar curve patterns according to present knowledge have to be treated differently [126]. Until now there is no evidence for the treatment of lumbar Scheuermann with physio-logic exercises, the sagittal profile of lumbar and thoracolumbar Scheuermann is not very different to that of patients with scoliosis. In the latter condition the physio-logic exercises have been shown to be beneficial and this is why there is good reason to assume they will also be beneficial for lumbar kyphoses without scoliosis [126]. Rehabilitation of Scheuermann's hyperkyphosis in international literature is regarded as being effective and specialist pattern-specific physiotherapy and bracing are the treatments of choice.

### 15.18 Hyperkyphosis in Adults

As recently as 2014, a review [162] highlighted the lack of treatment protocol and guidelines and evidenced-based treatments for hyperkyphosis in the elderly population. The authors state that despite the significant reported effects on function, quality of life and mortality [161-167], a negative impact on gait and balance and increases the risk of falls and fractures [167-172] there seems to be no definitive treatment for this age-group. The growing aging population and the financial impact on struggling health systems has not been calculated in the literature, but is likely to be significant and possibly comparable to other diseases and long-term conditions with more media coverage and more invested interest in research. The etiology is complex as with most cases in the elderly population there are multiple factors involved with aging and the spine, but the most commonly reported symptoms are similar to that of the adolescent group; weak back extensor muscle strength, loss of spinal mobility and pain. Alongside the general degenerative components such as disc dehydration and changes in vertebral shape/wedging [97, 162, 173].

Treatments include surgery, bracing, taping and physiotherapy, even though surgery, like in scoliosis surgery is not always indicated in age-related hyperkyphosis due to a lack of evidence and a high risk in this elderly population [97, 162]. Spinal orthoses have been used to reduce kyphosis; however, they have only been tested in women with underlying spinal osteoporosis [174]. In the ageing population, the physiologic brace can be used to reduce pain and provide an improvement on posture in functional activities [134].

In contrast to surgery treatment options, exercise allows individuals with hyperkyphosis to take an active role and keep their back and body fit in older age. Other health benefits have been reported in exercise-based treatment in the older adult [97]. Exercises that involve flexion tend to increase the risk of fractures whereas those that involve extension have reduced risk of vertebral fracture. ‘Prone-trunk lift’ [175, 176] exercises that aim on stretching the back extensors in a gravity resisted position have an RCT and long-term follow-up to back the effectiveness. Although, most frail patients with respiratory issues, common amongst hyperkyphotic patients [166], rarely tolerate prone-lying due to worsening the collapse on the anterior rib cage and therefore adapting prone exercises to positions where the rib cage is free to expand, such as the physio-logic exercises outlined [150] maybe more easily tolerated by this patient group. Those exercise programs that involve Activities of Daily Living (ADL) and postural training have shown evidence of improvement [97, 173] in those vulnerable patients in the community. As adults are more likely to spend more time seated and lying, posture and ADL posture is even more essential to address in this adult population.

Specifically, exercises that aim to increase back extensor strength and spinal flexibility may decrease hyperkyphosis and, if combined with postural training, may enable older adults to maintain a more upright posture. All the exercise interventions identified in the review [97], all of these studies included exercises aimed at improving back extensor strength suggests that there is some consensus among researchers in the field and that this is an important component of exercises for this patient group. Out of all recent reviews on the literature, the 2 high-quality RCTs that incorporated physical therapy into their interventions observed a statistically significant improvement in kyphosis within the intervention group [164, 177].

The literature in this area of research is of a poorer quality than that in the adolescent group; reasons for this can be the difficulties in excluding significant medical histories from the older adult population samples and difficulties with defining a relevant pre and post intervention outcome measure [162]. Further RCT’s in multiple centers will add to the current body of research, but currently the use of exercise has more evidence with adults with hyperkyphosis than professionals in this area may think. Further research using well established sagittal pattern-specific programs by specialist trained therapists in this patient age group should add to this already growing body of research. The other consideration is that, like with many things, the older adult does not always actively search for exercise-based treatment as much as the younger population and the professionals working in this area may give up too easily on these patients, or lack the specialist training, at post-graduate level, to address this. More focus and open discussions in general media and health professional platforms, may help highlight the need for further research into these conditions and the impact they have on both children and adults, may also provide more drive to invest in such research for the future.

### 15.19 Evidence for Braces in Adults with Spinal Deformities

A systematic review by [178] looked for high quality evidence regarding the orthotic treatment of adult onset spinal deformities. The search produced no valid papers, but did reveal one potentially relevant ongoing study related to scoliosis [179]. Four papers were found and reviewed relating to the orthotic treatment of kyphosis in subjects of over 60 years of age which reported positive results related to balance scores, reduction of deformity, muscle strength and pain. All four studies had inherent weaknesses in study design, and to various extents internal and external validity. Given this and the small number of reports discovered the results should be viewed with caution.

### 15.20 Surgery

Newer surgical techniques used in the treatment of kyphosis include vertebroplasty and kyphoplasty [180]. In the former procedure, the vertebrae are injected with a bone cement to improve the strength of the weakened bone. In kyphoplasty, cement is injected after reducing the wedging by inflating a balloon inside the vertebral body itself and filling the void produced with cement [180]. Both of these procedures require at least sedation and local anaesthesia but sometimes require general anaesthesia. Both operations can be performed using only very tiny incisions under x-ray direction.

## CONCLUSION

Currently, there is level I evidence in support of scoliosis specific exercises and bracing in the conservative treatment of AIS. It is interesting to note that the available evidence supports the use of PSSE and suggests that AIS patients should be treated with both PSSE and bracing. For patients with curves exceeding the formerly assumed range of conservative indications, conservative treatment should perhaps also be considered [83], given that there is an absence of long-term evidence of serious medical consequences with non-surgical management of curves ≥40^o^ and that there are no meaningful clinical significant differences in SRS-22r scores at average 8 year follow-up between AIS patients with curves ≥40^o^ treated with or without surgery [83].

For adolescents with hyperkyphosis and adults with scoliosis and hyperkyphosis, there is at present, a lack of evidence in support of either conservative or surgical treatments. Surgery should be considered with caution, as it is associated with a number of long-term complications [107, 177, 178, 181, 182].
